# Supplementary material for: Transient Agarose Spot (TAS) Assay: A New Method to Investigate Cell Migration
Source: Int J Mol Sci. 2022 Feb 14;23(4):2119. doi: 10.3390/ijms23042119 (PMC8880674; doi:10.3390/ijms23042119)
Supplement: Supplementary file 1 [file ijms-23-02119-s001.zip › ijms-1542157-SI.pdf]

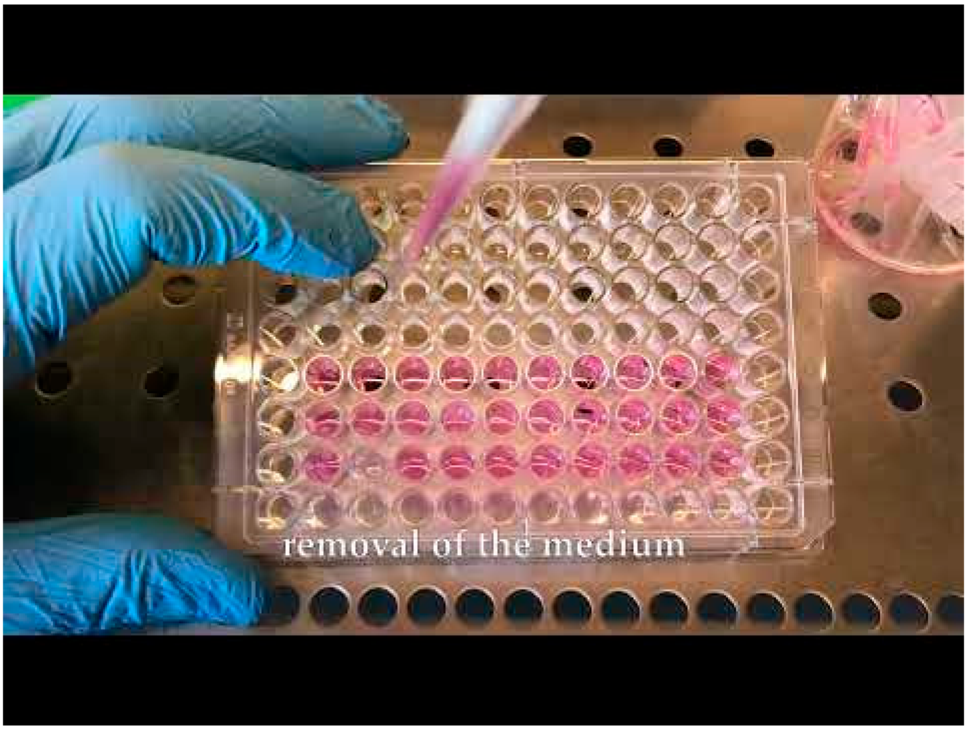

Video S1. Instructional video (https://youtu.be/nAI1QWpJrAI)

Table S1. Human genes involved in fibroblast-related biological processes based on Gene Ontology (GO) database (release date 2021-12-15).

GO term	Gene
fibroblast migration (GO:0010761)	*TGFB1, HAS1, PTK2, CCN3, AKT1, ZFAND5, CYGB, PIP5K1A, PAK3, ARHGAP4, THBS1, PML, FGF2, ARHGEF7, TMEM201, DDR2, PDLIM1, RAC1, IQGAP1, PLEC, HYAL2, PRKCE, ITGB1BP1, ILK, DMTN, LAMTOR2, SGPL1, BAG4, ITGB1, ACTA2, MACIR, SLC8A1, ARID5B, hsa-miR-145-5p, hsa-miR-124-3p, hsa-miR-146b-5p, AKAP12*
fibroblast proliferation (GO:0048144)	*PEX2, PDGFB, MYC, NF1, PARP10, WAPL, NGFR, AGT, MED25, CDK4, CDK6, LTA, CCNB1, CDC6, DAB2IP, ESR1, FOSL2, LIG4, NUPR1, MED31, DHX9, GSTP1, SKI, COL3A1, PDGFC, NDUFS4, PAWR, FBXO4, PML, E2F1, DDR2, JUN, C1QL4, WNT5A, TP53, WNT1, PDGFRA, CDKN1A, S100A6, SFRP1, RNASEH2B, EREG, EMD, IGF1, CDC73, MORC3, PLA2G1B, IFI30, BMI1, CTNNB1, FGF10, CD74, WNT2, PDGFD, FTH1, MIF, AQP1, FN1, CTC1, WNT7B, SOD2, IL13, SPHK1, PDGFRB, PDGFA, NLRC3, TRIM32, BAX, SIRT6, CD300A, CCNA2, ZMIZ1, DACH1, ZMPSTE24, TP53INP1, EGFR, ABL1, B4GALT7, hsa-miR-17-3p, hsa-miR-494-3p, hsa-miR-155-5p, hsa-miR-124-3p, GAS6*
extracellular matrix organization (GO:0030198)	*TGFB1, LOX, HAS2, MMP19, HAS1, CTSK, LOXL2, ERCC2, MPZL3, NF1, COL8A1, HSD17B12, IER3IP1, CARMIL2, GSN, LAMC1, TGFBI, TNXB, POMT1, SOX9, CCN1, PHLDB2, LAMB2, NTN4, COL1A2, SPOCK2, RIC1, COL16A1, COL4A4, C6orf15, COL9A1, CST3, AGT, ADAMTS14, EGFLAM, TLL2, NFKB2, COL28A1, CTSG, ADAMTS18, LAMB3, ITGA8, IBSP, FKBP10, LAMB4, SERPINB5, ADAMTSL4, LUM, NPNT, VIT, TNF, HPN, MMP12, COL12A1, FOXC2, COL22A1, HPSE2, TLL1, CRTAP, ANGPTL7, MMP28, MPV17, LTBP3, ADAMTS8, THSD4, MMP26, ST7, ADAMTSL5, COL4A5, RUNX1, SPINT2, COMP, CCN2, CLASP2, SERAC1, FLOT1, MMP25, ENG, MMP23B, ATXN1L, WT1, COL24A1,*

MMP10, MMP7, TNFRSF1A, FAP, PLOD3, GREM1, COL4A1, COL3A1, COL2A1, COL1A1, FERMT1, IMPG1, NPHS1, FBLN2, BMP2, KLK7, ADAMTS5, RGCC, LOXL4, FGFR4, CPB2, GAS2, ADAMTS10, PRSS2, CYP1B1, MIA, LAMB1, MMP8, FMOD, NID2, ANTXR1, VWA1, COL11A1, FSCN1, ADAMTS6, ADAMTS7, MMP20, SPINK5, RIC8A, TNFRSF1B, DDR2, MARCOL, IMPG2, MMP9, COL4A2, TMPRSS6, CHADL, TGFBR1, COLGALT2, EXOC8, PDPN, HAS3, PXDN, CRISPLD2, MMP16, MMP15, LOXL3, COL10A1, POSTN, RXFP1, COL6A5, SCX, QSOX1, CSGALNACT1, TNFRSF11B, APLP1, PDGFRA, ADAMTS19, ADAMTS15, ADAMTS16, ADAMTS17, ADAMTS12, MMP21, TNR, COL14A1, P3H4, CAV2, COL23A1, MMP2, MMP3, MFAP4, TGFB2, ECM2, ELANE, CLASP1, FBLN1, KIF9, EGFL6, AEBP1, PHLDB1, ELF3, TPSAB1, RB1, COL13A1, NOX1, TCF15, SERPINH1, GFAP, CAPG, HAPLN2, BMP1, OTOL1, SMPD3, B4GALT1, MYH11, DAG1, SLC39A8, LCP1, VIPAS39, CFLAR, LRP1, SERPINF2, SPINT1, ABI3BP, DPT, WDR72, MELTF, RAMP2, MYO1E, FOXF2, FOXF1, COL6A6, COL11A2, COL9A2, COL9A3, P4HA1, ADAM10, GPM6B, ADAM15, CAV1, CTSV, PRDX4, OLFML2B, OLFML2A, ADAMTS13, CCDC80, MMP11, NOTCH1, MYF5, PTX3, ADAMTSL2, FURIN, WNT3A, KAZALD1, COL4A3, DMP1, CMA1, MMP14, LOXL1, DDR1, ADAMTS3, ADAM8, NID1, SH3PXD2B, TMEM38B, MMP13, NTNG2, EMILIN1, COLQ, OPTC, SMAD3, COLGALT1, MMP27, ELN, CTSS, ADTRP, IL6, PRDM5, FLRT2, PBXIP1, KLK5, ADAMTS2, COL5A3, NTNG1, PAPLN, SULF1, SULF2, MMP24, KLK4, COL19A1, COL18A1, COL15A1, ADAMTS20, EXT1, RECK, EFEMP2, COL5A2, PLG, PRSS1, HMCN1, NR2E1, CREB3L1, SLC2A10, ITGB1, GFOD2, ATP7A, NOXO1, COL17A1, COL5A1, TNXA, ABL1, BCL3, DPP4, ADAMTS9, COL8A2, DNAJB6, ADAMTS4, ADAMTS1, COL4A6, TIE1, IHH, ERO1A, SFRP2, ADAMTSL3, FBLN5, ZNF469, SMOC2, MMP17, NDNF, hsa-miR-98-5p, hsa-miR-29b-3p, tenascin-x_human, COL27A1, WASHC1, GAS6, SMOC1, MMP1, APP, FOXC1

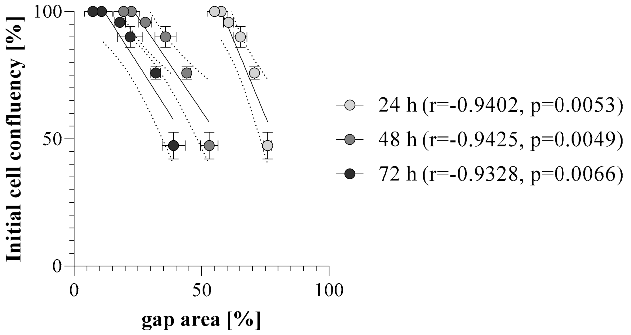

Figure S1. Correlation of cell density and gap closure. To determine optimal cell count, TAS migration assay was performed on MRC-5 cells. Correlations between initial cell confluency and gap areas at 24, 48 and 72 hours were determined by Pearson's coefficients (r).

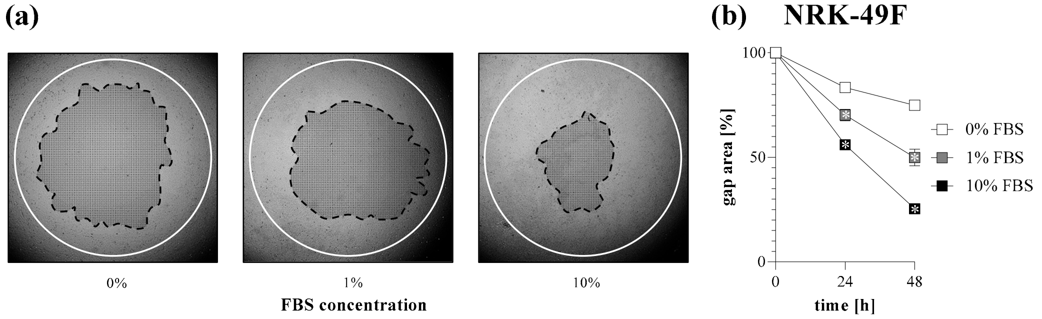

Figure S2. Effect of FBS treatment on cell migration of renal fibroblasts. To determine the effect of serum addition, TAS assay was performed on NRK-49F cells. (**a**) Cell-free zone areas were analysed graphically after brightfield microscopy. Lines in representative images indicate the gap edges at 0 (white) and 48 (black) hours after gel removal. (**b**) The gap closure was monitored for 48 hours after gel removal. Results are presented as mean ± SD (*n* = 8). * $p < 0.05$ vs. 0% FBS at the concerning time (two-way ANOVA).

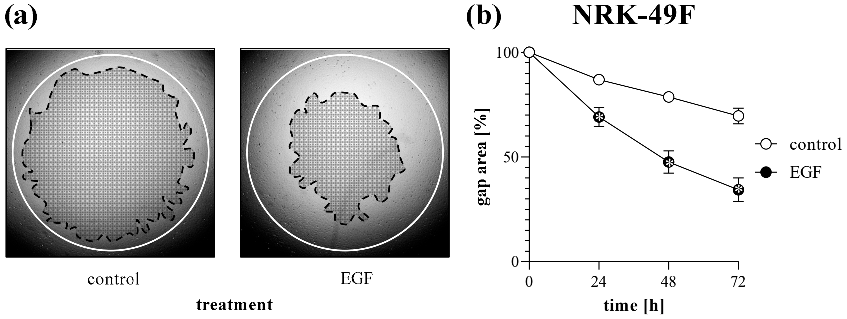

Figure S3. Effect of growth factor treatment on cell migration of renal fibroblasts. To determine the effect of EGF treatment, TAS assay was performed on NRK-49F cells. (**a**) Cell-free zone areas were analysed graphically after brightfield microscopy. Lines in representative images indicate the gap edges at 0 (white) and 48 (black) hours after gel removal. (**b**) The gap closure was monitored for 72 hours after gel removal. Results are presented as mean ± SD (*n* = 8). * $p < 0.05$ vs. 0% control at the concerning time (two-way ANOVA).

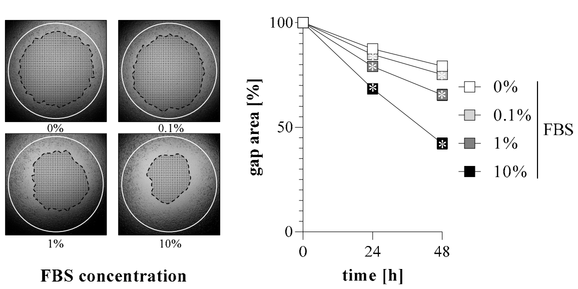

Figure S4. Effect of FBS treatment on wound healing of lung epithelial cells. To determine the effect of serum addition, TAS assay was performed on A549 cells. (**a**) Cell-free zone areas were analysed graphically after brightfield microscopy. Lines in representative images indicate the gap edges at 0 (white) and 48 (black) hours after gel removal. (**b**) The gap closure was monitored for

48 hours after gel removal. Results are presented as mean ± SD (n = 8). * p < 0.05 vs. 0% FBS at the concerning time (two-way ANOVA).

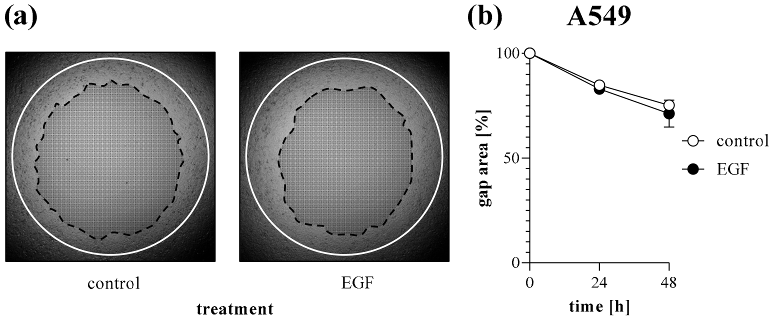

Figure S5. Effect of growth factor treatment on wound healing of lung epithelial cells. To determine the effect of EGF treatment, TAS assay was performed on A549 cells. (**a**) Cell-free zone areas were analysed graphically after brightfield microscopy. Lines in representative images indicate the gap edges at 0 (white) and 48 (black) hours after gel removal. (**b**) The gap closure was monitored for 48 hours after gel removal. Results are presented as mean ± SD (n = 8). * p < 0.05 vs. 0% control at the concerning time (two-way ANOVA).

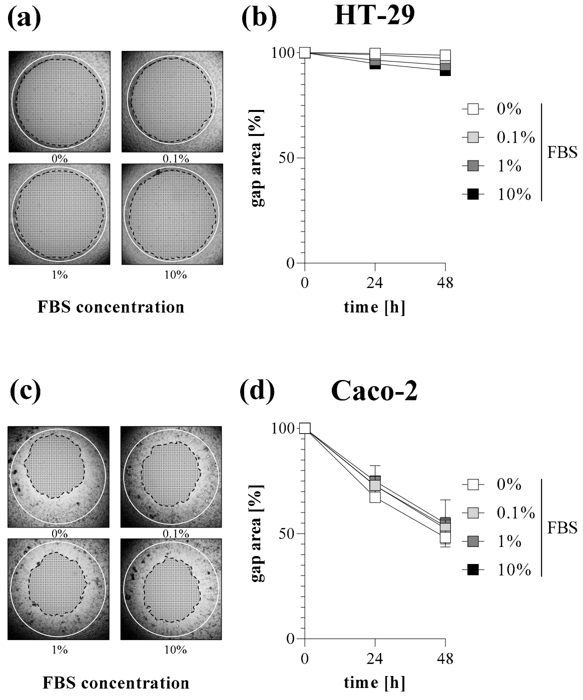

Figure S6. Effect of FBS treatment on the invasion of cancer cells. To determine the effect of serum addition, TAS assay was performed on (**a**, **b**) HT-29 and (**c**, **d**) Caco-2 cells. (**a**, **c**) Cell-free zone areas were analysed graphically after brightfield microscopy. Lines in representative images indicate the gap edges at 0 (white) and 48 (black) hours after gel removal. (**b**, **d**) The gap closure was monitored for 48 hours after gel removal. Results are presented as mean ± SD (n = 8). * p < 0.05 vs. 0% FBS at the concerning time (two-way ANOVA).

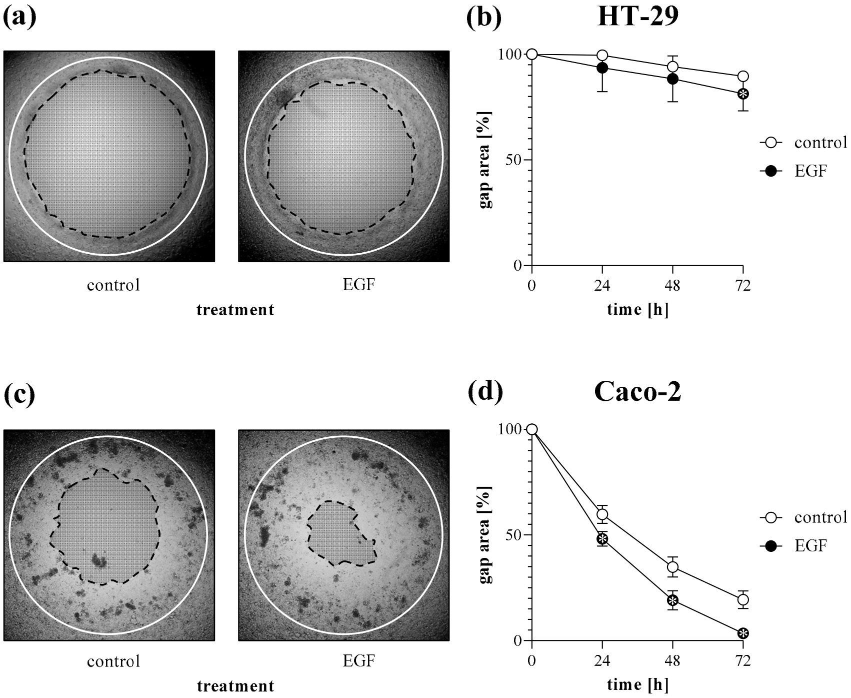

Figure S7. Effect of growth factor treatment on the invasion of cancer cells. To determine the effect of EGF treatment, TAS assay was performed on (**a**, **b**) HT-29 and (**c**, **d**) Caco-2 cells. (**a**, **c**) Cell-free zone areas were analysed graphically after brightfield microscopy. Lines in representative images indicate the gap edges at 0 (white) and 48 (black) hours after gel removal. (**b**, **d**) The gap closure was monitored for 72 hours after gel removal. Results are presented as mean ± SD (*n* = 8). *p<0.05 *vs.* 0% control at the concerning time (two-way ANOVA).

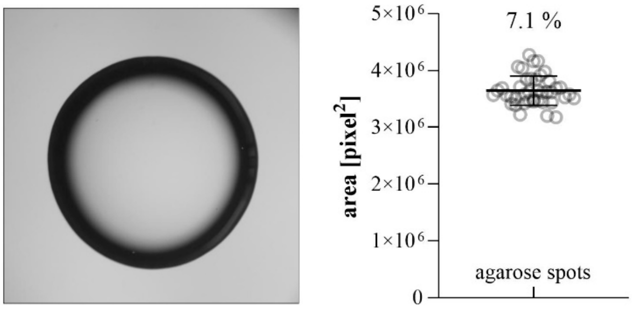

Figure S8. Reproducibility agarose dropping. Consistency of agarose spot sizes were determined by graphical analyzis (*n* = 40). Percentage values indicate the coefficient variation of received area.
